# Surveillance of *Escherichia coli* clones and their antimicrobial resistance profiles in wastewater and drinking water treatment plants of Barcelona, Spain

**DOI:** 10.1371/journal.pone.0355125

**Published:** 2026-07-31

**Authors:** Victoria Ballén, Enmanuel Cornielle, Anna Pinar-Méndez, Carles Vilaró, Belén Galofré, Sara Martí, Aida González-Diaz, Sergi Sanz, Aniol Vilamala, Sara M. Soto

**Affiliations:** 1 ISGlobal, Barcelona, Spain; 2 Faculty of Pharmacy and Food Sciences, University of Barcelona (UB), Barcelona, Spain; 3 Aigües de Barcelona, Empresa Metropolitana de Gestió del Cicle Integral de l’Aigua, Barcelona, Spain; 4 Microbiology Department, Hospital Universitari Bellvitge, IDIBELL-UB, L’Hospitalet de Llobregat, Spain; 5 Research Network for Respiratory Diseases (CIBERES), ISCIII, Madrid, Spain; 6 Consorcio de Investigación Biomédica en Red de Epidemiología y Salud Pública (CIBERESP), Madrid, Spain; 7 Department of Basic Clinical Practice, Faculty of Medicine, University of Barcelona, Barcelona, Spain; 8 CIBER Enfermedades Infecciosas (CIBERINFEC), Instituto de Salud Carlos III, Madrid, Spain; 9 Faculty of Medicine and Health Sciences, University of Barcelona (UB), Barcelona, Spain; Faculty of Veterinary Medicine, Qena University, EGYPT

## Abstract

Antimicrobial resistance (AMR) is a major global health threat, and environmental reservoirs such as wastewater (WWTPs) and drinking water treatment plants (DWTPs) may facilitate its persistence and spread despite reducing bacterial loads. We investigated 152 antibiotic-resistant *Escherichia coli* strains collected across treatment stages from two WWTPs and one DWTP in Barcelona, Spain. Strains were characterized through antimicrobial susceptibility testing, whole-genome sequencing, multilocus sequence typing, biofilm assays, and screening of antimicrobial resistance genes (ARGs), virulence factors (VFGs), biocide and heavy-metal tolerance genes (HMTGs). Although *E. coli* bacterial loads decreased along treatment, AMR remained highly prevalent: 85.5% of strains were multidrug-resistant (MDR), 5.3% extensively drug-resistant, and 11.2% carbapenemase-producers. Strains harboring integrase genes were 2.4 to 11.8 times more likely to harbor ARGs for sulfonamide, aminoglycoside, phenicol, trimethoprim, mercury and quaternary-ammonium compounds. Strains carrying *bla*_CTX-M_ genes were 3.0 to 20.3 times more likely to carry VFGs, while high-risk clones were 3.2 to 7.0 times more associated with VFGs. Some MDR and high-risk *E. coli* clones persisted in reclaimed water, and one MDR strain was detected at the DWTP inlet. These findings highlight environmental AMR reservoirs as a public health concern and support a One Health approach integrating antibiotic stewardship and environmental monitoring.

## Introduction

Wastewater treatment plants (WWTPs) receive effluents from domestic, industrial, agricultural, and hospital sources, creating a complex mix of pollutants such as antibiotics, heavy metals, and biocides. This chemical load may exert selective pressure on microbial communities and may promote the survival of antimicrobial-resistant bacteria (ARBs), facilitating the horizontal gene transfer of antimicrobial resistance genes (ARGs) through mobile genetic elements like plasmids, transposons, and integrons [1–4]. Within the WWTPs, biofilms could act as dynamic reservoirs for antimicrobial resistance (AMR), where dense microbial interactions may increase the genetic exchange at rates far exceeding those observed in planktonic populations [5].

Although WWTPs effectively reduce organic matter, nutrients and microorganisms [6], they are not explicitly designed to eliminate ARBs or ARGs [4]. Thus, ARBs and ARGs are introduced by treated effluents into downstream ecosystems, which may have an impact on rivers, lakes, coastal areas, and ultimately may endanger environmental and human health [7]. Recent studies identify WWTPs as sources of pathogenic and multidrug-resistant (MDR) bacteria, especially extended-spectrum beta-lactamase (ESBL)-producing *Enterobacteriaceae* like *E. coli*, and bacteria resistant to last-resort antibiotics such as carbapenems or colistin (COL) [8–10]. The World Health Organization classifies these ARBs as high-priority pathogens, with clear implications for public health [11].

Drinking water treatment plants (DWTPs) use multi-stage treatment processes such as grit removal, coagulation/flocculation, sedimentation, sand or carbon filtration, and, in most cases, disinfection to ensure potable water [12]. While highly effective at ensuring water quality, DWTPs may not fully eliminate antibiotics, ARBs, or ARGs, similarly to WWTPs, and thus may act as hotspots where AMR may persist and potentially disseminate into downstream environments [13,20].

From a One Health perspective, addressing these challenges requires integrated approaches. In this context, *E. coli* holds particular significance. It acts as a fecal indicator, a microbiological model and a potential and versatile pathogen. Pathogenic strains include extra-intestinal pathogenic *E. coli* (ExPEC), responsible for urinary tract infections (UTIs), neonatal meningitis, and sepsis, and diarrheagenic *E. coli* (DEC), implicated in gastrointestinal diseases [8,14].

The emergence of high-risk MDR *E. coli* clones reported in some studies in both the environment and clinical settings is also concerning. For instance, ST131 and ST69 have been frequently identified in raw sewage and river waters in the Barcelona region, showing virulence profiles similar to clinical strains [15]. Other studies report the repeated isolation of ST131 and ST648 from wastewater, indicating that these clones, including those with *bla*_CTX-M-15_, are common and may contribute to community-derived or persistent contamination [16]. Recently, an investigation conducted throughout Catalonia found ESBL-producing *E. coli* in 31 WWTPs influent and effluent (18.5% and 12.8%, respectively), with ST131 and ST162 being the most prevalent clones [17]. Importantly, it has also been documented that MDR *E. coli* carrying ESBL plasmids can reach drinking‑water supplies. One study in France isolated a sequence type (ST) 48 *E. coli* harboring a *bla*_CTX-M-1_ IncI1/ST3 plasmid from treated drinking water, demonstrating that water intended for human consumption can act as a reservoir and potential route for human exposure to ESBL genes, even in high‑income countries [18].

Due to its widespread presence, genetic adaptability, and clinical relevance, *E. coli* is a great indicator for studying AMR dynamics in aquatic ecosystems [12]. Characterizing MDR and high-risk *E. coli* clones from WWTPs and DWTPs is essential to assess ecological and health-related risks and to guide targeted interventions for the control of AMR [19].

In response to these concerns, this study provides a comprehensive genomic and phenotypic characterization of AMR *E. coli* strains across treatment stages in two WWTPs and one DWTP in Barcelona, combining whole-genome sequencing, phenotypic assays, and statistical modeling to elucidate persistence mechanisms and co-selection patterns.

## Materials and methods

### Sampling and strains isolation procedures

Four sampling campaigns were carried out in 2023, two in winter and two in summer, at two WWTPs and one DWTP located in the metropolitan area of Barcelona, Spain. At Baix Llobregat WWTP, samples were collected from the primary inlet, secondary outlet, tertiary outlet, and advanced tertiary outlet. At Gavà-Viladecans WWTP, sampling points included the primary inlet, the secondary outlet from the Integrated Fixed-Film Activated Sludge (IFAS) system, the secondary Membrane BioReactor (MBR) outlet, and the tertiary outlet. At the Sant Joan Despí DWTP, samples were taken from the inlet and the outlet. Ethics approval was not required, because only environmental samples were analyzed.

Water samples were processed using two approaches based on sampling point and matrix complexity. For inlet waters and the primary and secondary treatment stages of the WWTPs, 1 L samples were collected and processed directly without concentration. In contrast, samples from the secondary MBR, tertiary and advanced tertiary stages of the WWTPs were concentrated from 100 L using the Rexeed™ 25-A ultrafiltration system. For the DWTP outlet, 1,000 L were concentrated using the same system. Ultrafiltrated samples were then reduced to a final volume of 2–6 mL before analysis.

The overall methodology for isolating ARBs has been described in our previous report [20], where all ARBs recovered were characterized. In the present study, however, the analysis was specifically focused on the resistant *E. coli* strains collected in this first study. Briefly, 50 µL of each sample (direct or concentrated) and serial dilutions (10^−1^ and 10^−2^) were inoculated onto in-house prepared LB agar plates (Condalab, Madrid, Spain), supplemented with different antibiotics, as well as onto two commercial chromogenic media: CHROMID® ESBL and CHROMID® CARBA SMART (bioMérieux, Marcy-l’Etoile, France). Plates were incubated at 35°C ± 2°C for 18–24 hours. Colonies were subcultured on Columbia agar with 5% sheep blood (Beckton Dickinson, New Jersey, USA) and identified by matrix-assisted laser desorption ionization time-of-flight mass spectrometry (MALDI-TOF/MS) (MALDI Biotyper, Bruker Daltonik GmbH, Bremen, Germany).

### Antibiotic susceptibility testing

*E. coli* strains were assessed for antibiotic susceptibility through the disk diffusion method, following Clinical and Laboratory Standards Institute (CLSI) guidelines [21]. Susceptibility testing was performed for 13 antibiotics (Beckton Dickinson, New Jersey, USA), including amikacin (AN, 30 µg), ampicillin (AMP, 10 µg), cefepime (FEP, 10 µg), cefotaxime (CTX, 30 µg), ceftazidime (CAZ, 30 µg), chloramphenicol (CHL, 30 µg), ciprofloxacin (CIP, 5 µg), gentamicin (GM, 10 µg), imipenem (IPM, 10 µg), meropenem (MEM, 10 µg), piperacillin-tazobactam (TZP, 10 µg), trimethoprim-sulfamethoxazole (SXT, 1.25/23.75 µg), and tetracycline (TET, 10 µg). In addition, COL susceptibility was assessed using the broth microdilution method, following CLSI guidelines. *E. coli* ATCC 25922 was used as the quality control strain.

Furthermore, phenotypic identification of ESBL and carbapenemases was conducted following CLSI guidelines [21]. Strains identified as ESBL and carbapenemase producers were subsequently tested using the NG-Test®/CTX-M Multi and NG-Test®DetecTool CARBA 5 kits (NG-Biotech Laboratories, Guipry, France), respectively, following the manufacturer’s instructions.

### Whole-Genome Sequencing (WGS) and bioinformatic analysis

All 152 confirmed *E. coli* strains resistant to at least one antibiotic family underwent WGS. Genomic DNA was extracted using the MagMAX™ DNA Multi-Sample Ultra 2.0 Kit on a KingFisher Flex instrument (Thermo Fisher Scientific, Waltham, USA) and quantified with a Qubit Flex Fluorometer (Thermo Fisher Scientific, Waltham, USA). Library preparation was performed with the DNA Prep Library Preparation Kit, and sequencing was conducted on the Illumina NextSeq platform (2 × 150 bp, San Diego, USA). Data quality control and genome assembly were carried out using the Bactopia pipeline (https://github.com/bactopia/bactopia). *In silico* multilocus sequence typing (MLST) and serotyping were performed using the Bactopia MLST module, and ARGs were detected using the AMRFinder tool [22].

A phylogenetic tree was constructed using the alignment generated by Snippy and IQ-TREE, with the GTR + F + I + G4 model and 1,000 UFBoot replicates. The tree was midpoint-rooted, and *E. coli* K12 (NC_000913.3) was used as the reference strain. The final tree and its associated metadata were visualized in iTOL (itol.embl.de). Reads were deposited in the European Nucleotide Archive (Accession code: PRJEB102514).

Integrase genes (*intI1*, *intI2* and *intI3*), and additional VFGs were screened, including siderophore-associated (*entB* and *irp1*), adhesins (*pgaA, fimH*), and toxins (*clbA, aggR*, *eltA, stxA1*). All these genes were mapped against reference sequences using Geneious Prime® v2025.1.3.

Assembled genomic DNA sequences of *E. coli* were analyzed using the Enterobase web-based platform (https://enterobase.warwick.ac.uk/). Phylogroups were determined using the Clermont Typing method, and MLST was assigned according to the Atchman 7-gene scheme for strains lacking a previously defined profile.

### Biofilm analysis

*E. coli* resistant strains were cultured overnight on Columbia agar with 5% sheep blood at 37°C. Biofilm production was determined using the protocol previously established in our laboratory [23]. Briefly, a single colony was inoculated into LB broth and incubated overnight at 37°C with shaking at 180 rpm. After incubation, the cultures were diluted 1:100 in 200 µL of M63 medium supplemented with 0.25% glucose and added to 96-well flat-bottom non-treated polystyrene microtiter plates (Nunc™ Edge 2.0, VWR International, Barcelona, Spain). Then, the plates were incubated at 30°C for 48 hours. After incubation, biofilm quantification was performed using a crystal violet staining technique. Biofilm formation was measured at 580 nm using a Microplate Spectrophotometer (EPOCH 2 microplate reader, BioTek, Winooski, VT, USA) and analyzed according to the protocol reported by Stepanović [24].

### Statistical analysis

Categorical variables were described using absolute and relative frequencies and compared using chi-square tests or Fisher’s exact tests, as appropriate according to the assumptions of each test. In specific cases, when comparing the means of continuous variables between two groups, the t-test was applied. To assess the association between independent variables and the outcome of interest, logistic regression models with Firth’s penalization were fitted to reduce small-sample bias or sparse data bias. All statistical analyses were performed using Stata, version 19.1 (StataCorp LLC, College Station, TX, USA). A two-sided p-value < 0.05 was considered statistically significant.

To correlate genomic profiles with phenotypic antimicrobial resistance rates, associations were evaluated via two-tailed Fisher’s exact tests comparing susceptible (S) versus non-susceptible (I + R) isolates, with association strengths reported as Odds Ratios (OR). To control for multiple comparisons, p-values were adjusted independently within each antibiotic family using the Benjamini-Hochberg False Discovery Rate (FDR) procedure. All analyses were performed using R (v.4.5.3).

## Results

### *E. coli* identification and distribution at the different treatment plants

A total of 152 strains were identified as *E. coli* using MALDI-TOF. Of these, 97 (63.8%) were isolated from the Baix Llobregat WWTP, 54 (35.5%) from the Gavà-Viladecans WWTP, and one (0.7%) from the Sant Joan Despí DWTP. Among them, 56 (36.8%) were isolated from water samples collected during the winter campaigns, whereas 96 (63.2%) were recovered during the summer sampling campaigns.

At the Baix Llobregat WWTP, ARBs gradually decreased across successive treatment stages, reflecting a progressive reduction in bacterial load. Specifically, 31 *E. coli* strains were identified in the primary inlet, 30 in the secondary outlet, 28 in the tertiary outlet, and eight in the advanced tertiary outlet.

Conversely, at the Gavà-Viladecans WWTP, there was an initial slight increase in ARBs from the primary inlet (23 *E. coli* strains) to the secondary IFAS outlet (30 strains), followed by a sharp decline at the secondary MBR outlet (one strain). No ARB strains were isolated at the final tertiary outlet, indicating effective bacterial removal by the end of the treatment process.

At the Sant Joan Despí DWTP, a single *E. coli* strain was detected at the inlet, while none were found at the outlet.

### Antibiotic susceptibility testing

**Fig 1:**
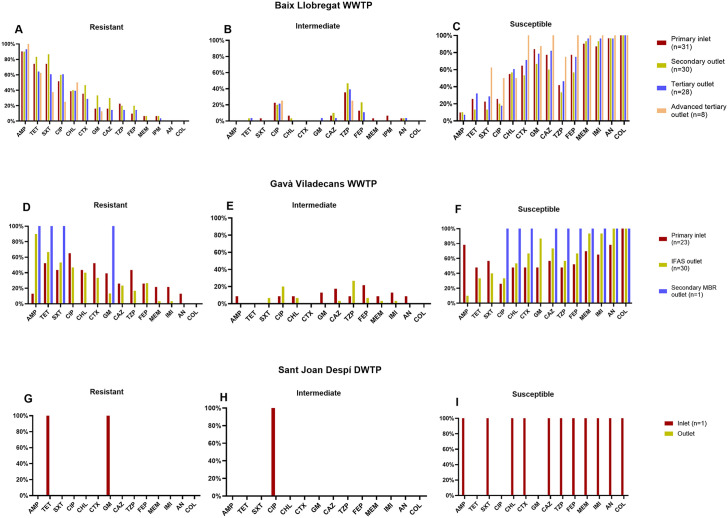
Overall antimicrobial resistance profiles of the AMR-*E. coli* strains isolated from WWTPs and DWTP. AM: Ampicillin; AN: amikacin; CAZ: ceftazidime; CHL: chloramphenicol; CIP: ciprofloxacin; COL: colistin; CTX: cefotaxime; FEP: cefepime; GM: gentamicin; IMI: imipenem; MEM: meropenem; SXT: trimethoprim/sulfamethoxazole; TET: tetracycline; TZP: piperacillin-tazobactam.

The overall antibiotic resistance profiles revealed a high prevalence of resistance to AMP (n = 138, 90.8%), followed by TET (n = 108, 71.1%), SXT (n = 96, 63.2%), and CIP (n = 82, 53.9%). Lower resistance rates were observed for CHL (n = 61, 40.1%), CTX (n = 55, 36.2%), GM (n = 36, 23.7%), TZP (n = 32, 21.1%), CAZ (n = 31, 20.4%), and FEP (n = 27, 17.8%). Resistance to carbapenems was low, with IPM and MEM both at 7.2% (n = 11). The lowest rate was observed for AN (n = 3, 2%), while no resistance to COL was detected. A summary of these results is shown in Fig 1.

Resistance profiles were categorized according to the criteria by Magiorakos *et al.* [25] revealing considerable variability among strains. Four strains (2.6%) exhibited resistance to only one antimicrobial category, while 10 strains (6.6%) showed resistance to two antimicrobial categories. The majority (130 strains, 85.5%) were classified as MDR, while eight strains (5.3%) met the criteria for extensively drug-resistant (XDR).

Both WWTPs exhibited similar antibiotic resistance profiles, with no substantial differences. The single strain isolated from the inlet of the DWTP was classified as MDR, exhibiting resistance to TET and GM and intermediate susceptibility to CIP.

### Phenotypic characterization of ESBL and carbapenemase-producing strains

A total of 44 strains (28.9%) were phenotypically identified as ESBL producers, of which 41 were confirmed as CTX-M producers using the NG-Test®/CTX-M Multi assay. The distribution of ESBL-producing strains varied between the WWTPs: 27 of 44 (61.4%) originated from Baix Llobregat WWTP and 17 (38.6%) from the Gavà-Viladecans WWTP. This difference may be explained by several causes: i) differences in the capacity of each treatment plant; ii) differences in treatments processes; and iii) the geographical areas covered by each WWTP and, therefore, the inclusion of hospital discharges. No ESBL-producing strains were detected at the DWTP, either at the inlet or outlet.

Seventeen strains (11.1%) with intermediate susceptibility or resistance to carbapenems were phenotypically identified as carbapenemase producers, and all were confirmed using the NG-Test® DetecTool CARBA-5 test. Of these, seven strains (41.2%) were isolated from the Baix Llobregat WWTP (primary inlet, secondary outlet, and tertiary outlet) and ten (58.8%) from the Gavà-Viladecans WWTP (primary inlet and IFAS secondary outlet). The detected carbapenemases included eight KPC producers, three OXA-48 producers, two NDM producers, and one VIM-producer. Additionally, three strains showed co-production of carbapenemases: two co-producing KPC and OXA-48, and one co-producing KPC and VIM. Notably, OXA-48 producers were not detected at Baix Llobregat WWTP, whereas NDM producers were absent from Gavà-Viladecans WWTP. No IMP-producing strains were detected at either WWTP. Similarly to ESBL-producing strains, no carbapenemase-producing strains were detected at the DWTP.

### Whole-Genome Sequencing

#### Multilocus Sequence Typing, O and H serotyping.

Across the 152 *E. coli* strains analyzed, 73 distinct STs were identified. The highest diversity was found in the Baix Llobregat WWTP, with 54 different STs; while the Gavà-Viladecans WWTP presented 33 different STs. Among these, only 15 STs were identified in both WWTPs (Table 1).

**Table 1:** Sequence types by WWTP and DWTP.

	Sequence type (No. strains)
**STs found in both WWTPs**	ST10 (10), ST48 (3), ST58 (7), ST69 (3), ST88 (7), ST95 (4), ST117 (2), ST131 (19), ST162 (4), ST398 (2), ST399 (3), ST401 (6), ST635 (2), ST648 (6), ST744 (8)
**STs found exclusively in WWTP Baix Llobregat**	ST12 (1), ST34 (1), ST38 (1), ST56 (1), ST90 (2), ST93 (1), ST155 (1), ST165 (1), ST167 (1), ST206 (2), ST212 (1), ST349 (2), ST362 (2), ST410 (2), ST448 (2), ST540 (3), ST542 (2), ST617 (1), ST641 (2), ST683 (2), ST949 (1), ST1049 (1), ST1086 (1), ST1196 (1), ST1284 (1), ST1434 (1), ST1485 (1), ST1494 (1), ST1564 (1), ST1652 (1), ST1702 (1), ST1722 (1), ST2008 (1), ST2279 (1), ST2325 (1), ST3580 (1), ST7401 (1), ST10955 (1), ST13823 (1)
**STs found exclusively in WWTP Gavà-Viladecans**	ST44 (1), ST156 (1), ST345 (1), ST457 (1), ST480 (1), ST609 (1), ST1079 (1), ST1244 (1), ST1324 (1), ST1421 (1), ST1602 (1), ST1642 (1), ST1674 (1), ST2064 (1), ST13499 (1), ST14224 (1), ST17968 (1), ST17973 (2)
**STs found exclusively in DWTP Sant Joan Despí**	ST976 (1)

DWTP: Drinking water treatment plant; ST, sequence type; WWTP: Wastewater treatment plants.

Clinically significant high-risk clones associated with MDR and enhanced virulence were particularly prevalent, representing 57.9% of all strains. The most frequent STs were ST131 (19 strains, 12.5%), ST744 (eight strains, 5.3%), ST88 and ST58 (seven strains each, 4.6%), and ST10, ST648 and ST401 (six strains each, 3.9%). Additional high-risk STs, including ST12, ST38, ST44, ST48, ST69, ST93, ST95, ST117, ST162, ST167, ST345, ST410, ST457, ST617, ST641, and ST1485, were detected at lower frequencies. The only strain recovered at the DWTP inlet belonged to ST976, which is not classified as a high-risk clone.

Among carbapenemase-producers, the high-risk *E. coli* clone ST401 O159:H34 was the most frequent, including three OXA-48 and two KPC producers.

Among the 19 *E. coli* ST131 strains, the predominant serotypes were O25:H4 (11 strains, 57.9%) and O16:H5 (n = 8, 42.1%), some of which were recovered from the tertiary and advanced tertiary treatment stages of the Baix Llobregat WWTP, corresponding to reclaimed water.

Two ST744 strains, one from the primary inlet and the other from the secondary outlet of the Baix Llobregat WWTP, collected during the second summer sampling, were genotypically identical, suggesting persistence through water treatment. Likewise, two ST131 strains, one from the Baix Llobregat secondary outlet and the other from the Gavà- Viladecans IFAS outlet, obtained during the first and second winter samplings, respectively, displayed identical genomic profiles. The presence of this high-risk clone in both WWTPs emphasizes its relevance within the local wastewater environment.

#### Phylogroup classification.

Phylogroup A was the most prevalent (n = 59, 38.8%), followed by B1 (n = 33, 21.7%), B2 (n = 25, 16.4%), D (n = 11, 7.2%), C (n = 11, 7.2%), F (n = 10, 6.6%), G (N = 2, 1.3%) and E (n = 1, 0.7%). Notably, phylogroup A has a striking association with HMTGs. Among the 51 strains carrying *mer* genes, 51% belonged to phylogroup A; while 75.8% of *sil*-positive and 76.7% of *pco*-positive strains were also classified into this group. Moreover, 11 phylogroup A strains co-harbored *mer*, *pco* and *sil*.

In contrast, phylogroup B2 strains, well known for their association with ExPEC, exhibited a high prevalence of VFGs. All strains harboring the *hlyA*, *cnf1*, or *ibeA* genes belonged to this group, along with those carrying the *sfa* (100%), *sat* (85.7%), and *pap* (42.9%) genes. Phylogroup B2 was also linked to clinically relevant ARGs, with 60% of strains carrying *bla*_CTX-M_ and 56% carrying *bla*_TEM_.

**Fig 2:**
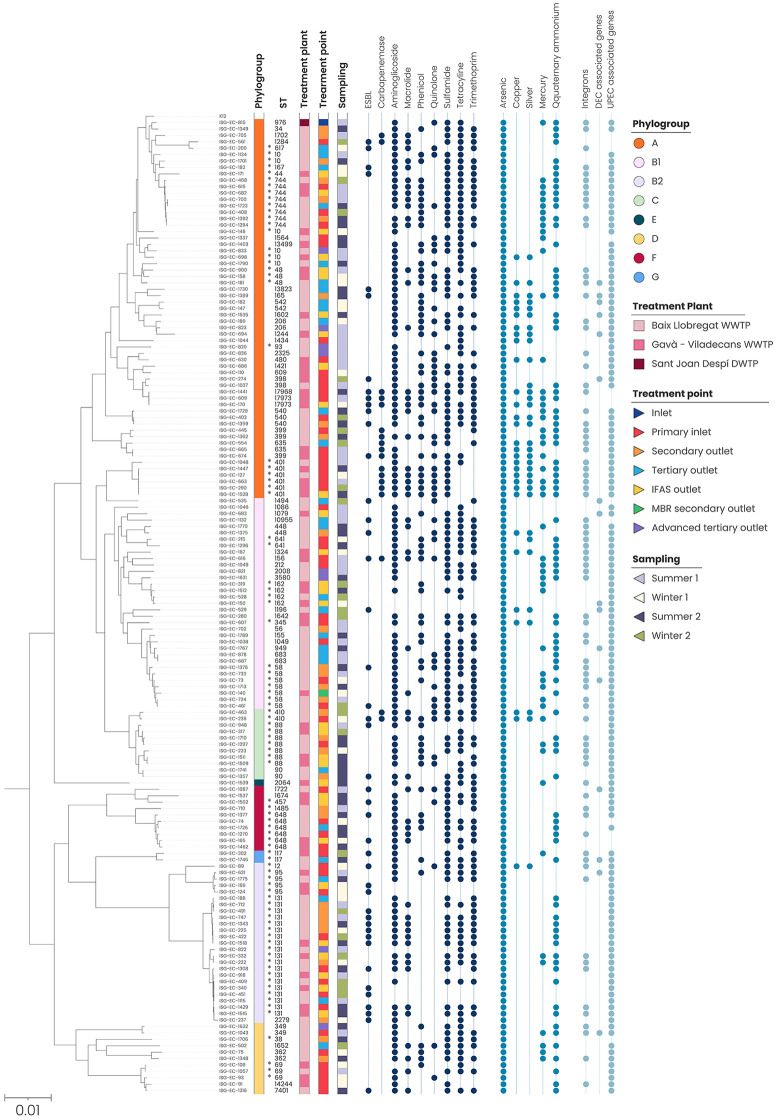
Phylogenetic tree of *E. coli* genomes. The tree illustrates their multilocus sequence type (ST), integrons, production of extended-spectrum β-lactamases (ESBLs) and carbapenemases, and the presence of antimicrobial resistance genes associated with different antibiotic families, genes associated with heavy metal and biocide tolerance, and genes associated with diarrheagenic *E. coli* (DEC) or urinary pathogenic *E. coli* (UPEC) pathotypes. Each wastewater treatment plant or drinking water treatment plant, treatment stage, and sampling campaign is indicated in separate columns and distinguished by color. A dot represents the presence of each gene.

The results of the shotgun sequencing are represented in Fig 2.

### Antimicrobial Resistance Genes (ARGs)

The prevalence of each ARG is summarized in Table 2.

**Table 2:** Distribution of AMR profiles, ARGs, VFGs, HMTG and biocide resistance genes by location.

	WWTP Baix Llobregat				WWTP Gavà			DWTP SJD
	Primary inlet (N = 31)	Secondary outlet (N = 30)	Tertiary outlet (N = 28)	Advanced Tertiary outlet (N = 8)	Primary inlet (N = 23)	Secondary IFAS outlet (N = 30)	Secondary MBR outlet (N = 1)	Inlet (N = 1)
**Resistance phenotype**								
**AN**	0	0	0	0	3	0	0	0
**AMP**	28	27	26	8	21	27	1	0
**FEP**	3	6	4	0	6	8	0	0
**CTX**	11	14	8	0	12	10	0	0
**CAZ**	5	9	4	0	6	7	0	0
**CHL**	12	12	11	4	10	12	0	0
**CIP**	16	18	17	2	15	14	0	0
**GM**	5	10	5	1	9	4	1	1
**IMI**	2	2	1	0	5	1	0	0
**MEM**	2	2	1	0	5	1	0	0
**TZP**	7	6	4	0	10	5	0	0
**SXT**	23	26	17	2	10	16	1	0
**TET**	23	25	18	8	12	20	1	0
**COL**	0	0	0	0	0	0	0	0
**ARGs**								
***bla***_**KPC**_	2	1	1	0	6	1	0	0
***bla***_**VIM**_	1	0	0	0	1	0	0	0
***bla***_**NDM**_	1	1	0	0	0	0	0	0
***bla***_**OXA-48/484**_	0	1	0	0	2	1	0	0
***bla***_***CTX-M-1***_	5	8	7	0	7	8	0	0
***bla***_***CTX-M-9***_	3	2	0	0	0	1	0	0
***bla***_**TEM**_	18	8	17	8	14	21	1	0
***bla***_**SHV**_	1	1	0	0	1	0	0	0
***bla***_**CMY**_	0	2	1	0	0	0	0	0
***bla***_**OXA**_	6	5	4	0	7	4	0	0
***bla***_**DHA**_	1	0	1	0	0	1	0	0
***bla***_**EC**_	31	30	28	8	23	30	1	1
***ble***	1	1	0	0	1	0	0	0
***mcr9.1***	1	0	0	0	2	0	0	0
***fosA***	1	0	0	0	0	0	0	0
***lnu***	2	3	4	1	2	3	0	0
***ermB***	1	1	1	0	0	1	0	0
***mef***	0	0	1	1	0	2	0	0
***mph***	11	14	10	0	8	7	1	0
***msr***	1	0	0	0	2	0	0	0
***arr-3***	1	1	1	0	5	2	0	0
***sat2***	0	1	0	0	3	3	0	0
***sul1***	18	18	11	2	10	8	1	1
***sul2***	19	18	9	4	7	10	1	0
***sul3***	3	5	4	2	2	4	0	0
***dfrA***	23	25	18	4	10	17	1	0
***qnrA1***	1	0	0	0	1	0	0	0
***qnrB***	3	1	4	0	1	2	0	1
***qnrS***	5	5	11	2	8	6	0	0
***aac(3)***	4	9	6	1	8	5	1	1
***ant(2´´)-Ia***	2	2	0	0	1	0	0	0
***ant(3´´)-Ia***	0	1	0	0	0	0	0	0
***aph(3´)***	15	19	8	4	11	15	1	0
***aph(4’)-Ia***	0	0	0	1	1	0	0	0
***aph(6)-Id***	12	16	7	3	10	14	1	0
***aadA***	19	20	18	7	11	15	1	1
***aac(6’)-lb-cr***	3	2	2	0	6	4	0	0
***aac(6’)-lb***	2	0	0	0	7	1	0	0
***catA1/catB3***	9	7	4	1	9	4	0	0
***cmlA***	4	2	3	2	2	3	0	0
***floR***	4	5	8	3	5	12	0	0
***tetA***	16	18	12	7	9	16	1	1
***tetB***	8	8	7	2	4	5	0	0
***tetM***	2	3	2	0	1	1	0	0
**VFGs**								
***iro***	5	5	5	2	4	6	0	1
***iuc***	11	14	12	1	7	12	1	1
***iutA***	11	13	12	1	7	12	1	1
***ybt***	19	19	8	3	9	13	1	1
***entB***	31	30	28	8	23	30	1	1
***irp1***	19	19	8	2	9	12	1	1
***pap***	4	5	4	2	5	1	0	0
***sfa***	1	0	0	0	1	0	0	0
***pgaA***	24	26	12	5	14	22	1	1
***fimH***	27	28	24	7	20	28	1	1
***cnf***	1	2	0	1	1	1	0	0
***hly***	1	2	0	1	1	1	0	0
***ibeA***	0	0	0	1	0	1	0	0
***clbA***	1	0	0	0	1	1	0	0
***astA***	3	1	5	0	2	4	0	0
***sat***	4	6	4	0	2	5	0	0
**HMTGs and Biocide resistance genes**								
***sil***	8	4	5	1	9	6	0	0
***mer***	12	11	6	3	10	8	0	1
***pco***	6	4	5	1	8	6	0	0
***ars***	31	30	28	8	23	30	1	1
***ter***	3	0	2	0	2	1	0	0
***qac***	21	21	16	4	13	11	1	1

AMP: Ampicillin; AN: amikacin; ARGs: antimicrobial resistant genes; CAZ: ceftazidime; CHL: chloramphenicol; CIP: ciprofloxacin; COL: colistin; CTX: cefotaxime; DWTP: drinking water treatment plant; FEP: cefepime; GM: gentamicin; HMTGs: heavy metal tolerance genes; IFAS: integrated fixed-film activated sludge; IMI: imipenem; MBR: membrane bioreactor; MEM: meropenem; N, number of strains; SXT: trimethoprim/sulfamethoxazole; TET: tetracycline; TZP: piperacillin-tazobactam; VFGs: Virulence Factor Genes; WWTP: wastewater treatment plant.

In terms of aminoglycoside resistance, the most frequent aminoglycoside resistance gene was *aadA*, followed by *aph(3’)* and *aph(6)-Id*.

All strains carried a *blaEC* family class C beta-lactamase gene. Among additional beta-lactamases, *bla*_TEM_ was the most prevalent, including variants *bla*_TEM-1_, *bla*_TEM-30_, *bla*_TEM-31_, *bla*_TEM-35_, *bla*_TEM-40_, *bla*_TEM-135_ and *bla*_TEM-136_.The *bla*_CTX-M-1_ group was the second most frequent, represented by *bla*_CTX-M-15_ (n = 26), *bla*_CTX-M-3_ (n = 5), *bla*_CTX-M-32_ and *bla*_CTX-M-55_ (n = 2 each). *bla*_OXA-1_ was identified in 24 strains, while the *bla*_CTX-M-9_ group was present in six strains, comprising *bla*_CTX-M-27_ (n = 4) and *bla*_CTX-M-14_ (n = 2). The three ESBL-producing strains not detected by the NG-Test®/CTX-M Multi assay were identified by sequencing as *bla*_SHV-12_ carriers.

All carbapenemases detected by NG-Test® DetecTool CARBA-5 were confirmed by sequencing. Among the 11 *bla*_KPC_-positive strains, one carried *bla*_KPC_, and 10 carried *bla*_KPC-2_. Additionally, five *bla*_OXA-48_, two *bla*_NDM-5_, and two *bla*_VIM-1_ positive strains were confirmed. Sequencing also revealed one *bla*_OXA-484_ carrying strain, increasing the total number of carbapenemase producers to 18. Co-occurrence of multiple beta-lactamase genes was frequent. For example, among the 24 *bla*_OXA-1_-positive strains, nine also carried *bla*_CTX-M-15_, six *bla*_KPC-2_, five *bla*_OXA-48_ and two *bla*_VIM-1_. Of the five *bla*_OXA-1_/*bla*_OXA-48_-positive strains, two additionally carried *bla*_FOX_ and *bla*_TEM_; one harbored *bla*_CTX-M-3_ and *bla*_KPC2_, another *bla*_CTX-M-15_ and *bla*_KPC-2_; and the remaining strain carried *bla*_FOX_.

In the case of colistin resistance, although no strains displayed phenotypic resistance, the *mcr9.1* was detected in three strains, one at the primary inlet of Baix Llobregat WWTP, and two at the primary inlet of Gavà-Viladecans WWTP.

Chloramphenicol O-acetyltransferase genes were identified in 37 strains: *catA1* (n = 21, 13.8%), c*atB3* (n = 13, 8.6%), *catA2* (n = 2, 1.3%), and *catB2* (n = 1, 0.7%). Two of these strains simultaneously carried *catA1/catB3*, and one carried *catB2/catB3*. Efflux genes *floR* (n = 36, 23.7%) and *cmIA* (n = 16, 10.5%) were also detected.

The most prevalent quinolone resistance gene was *qnrS*, followed by *qnrB*, and *qnrA*. One strain carried both *qnrB* and *qnrS* genes. *qepA* was only detected in one strain. *aac(6’)-Ib-cr5* gene, encoding an aminoglycoside 6’-N-acetyltransferase that also confers resistance to quinolones, was found in 15 strains, and the *aac(6’)-Ib-cr* gene in two strains.

Sulfonamide resistance genes included *sul1*, *sul2*, and *sul3*. Co-occurrence of *sul1/sul2* was observed in 30 strains (19.7%), *sul1/sul3* in three (2%), and *sul2/sul3* in eight strains (5.3%).

The most common TET resistance gene was *tetA*, followed by *tetB*, and *tetM*. Eleven strains (7.2%) carried multiple TET resistance genes: eight with *tetA*/*tetM*, and three with *tetA*/*tetB*.

**Phenotype-Genotype association** The genotype–phenotype associations between resistance genes and antibiotic non-susceptibility profiles are summarized in S1 File. The matrix is structured to contrast strong predictors against borderline trends and non-significant determinants across each antibiotic.

High-statistical relationships (p < 0.001) were found: i) GM resistance with the presence of *aac(3)* and *aac(6’)-Ib* genes; AMP resistance with the presence of *bla*_TEM_ gene; FEP and CTX resistance is associated with *bla*_CTX-M-1_ and *bla*_KPC_ genes; CAZ resistance with *bla*_CTX-M-1_ gene; CHL resistance is highly associated with the presence of both *cat*, *floR* and *cmlA* genes; CIP resistance is mainly associated with the presence of the *qnrS* gene; SXT resistance is associated with the presence of both *sul1*, *sul2*, *sul3*, and *dfr* genes; finally, TET resistance is highly associated with the presence of *tetA* and *tetB* genes.

#### Biocide and heavy metal tolerance genes.

Arsenic tolerance genes (*ars*) were present in all strains (100%), emphasizing their ubiquity across the strains. Mercury tolerance genes (*mer*) were present in 51 strains (33.6%), silver tolerance genes (s*il*) in 33 strains (21.7%), and copper tolerance genes (*pco*) in 30 strains (19.7%), while tellurium tolerance genes (*ter*) were less frequent, occurring in only eight strains (5.3%). Four strains (2.6%) carried all five classes of HMTGs, and 12 strains (7.9%) had the four most common (*mer/sil/pco/ars*). The *sil*/*pco* co-occurrence was common, detected in 30 strains (19.7%).

Quaternary ammonium compounds (QACs) tolerance genes were detected in 88 strains (58.0%) across all WWTP treatment stages and the DWTP inlet. Eight strains carried *qac* genes alongside the four most frequent HMTGs, and three strains carried *qac* genes with all five HMTGs.

#### Virulence Factor Genes.

WGS revealed a diverse repertoire of VFGs, including siderophores (*iro, iuc, iutA, ybt*), adhesins (*pap, sfa*), and toxins (*cnf1, hlyA, ibeA, sta, astA, eatA, sigA*). Additional VFGs, such as siderophore-associated (*entB* and *irp1*), adhesins (*pgaA, fimH*), and toxins (*clbA, aggR*, *eltA, stxA1*), were mapped against reference sequences using Geneious Prime® v2025.1.3. Overall, 96.7% of isolates carried at least one VFG.

**Siderophores:** The *entB* (enterobactin biosynthesis protein) gene was present in all strains (100%), *ybt* gene (yersiniabactin transport system) in 73 (48%), i*rp1* gene (yersiniabactin biosynthetic gene cluster) in 71 (46.7%), *iuc* gene (aerobactin synthesis proteins) in 59 (38.8%), *iutA* gene (aerobactin receptor) in 58 (38.2%), and *iro* gene (salmochelin biosynthesis proteins) in 28 (18.4%).

**Adhesins:** *fimH* (type 1 fimbrial adhesin) was the most prevalent, detected in 136 strains (89.5%). The *pgaA* (poly-N-acetylglucosamine export porin) gene occurred in 105 strains (69.1%), while *pap* (P fimbriae) and *sfa* (S fimbriae) genes were less frequent, detected in 21 (13.8%) and two strains (1.3%), respectively. These three adhesins (*fimH*, *pap* and *sfa*) are commonly linked to UTIs, whereas the *pgaA* gene is associated with biofilm formation. Only five strains lacked adhesin genes.

**Toxins:** DEC-associated toxin genes included *astA* (heat-stable enterotoxin EAST1), which was found in 15 strains (9.9%) from different treatment points, including the tertiary outlet of the Baix Llobregat WWTP; *eatA* (serine protease autotransporter toxin EatA) was detected in one strain from the tertiary outlet of the Baix Llobregat WWTP (0.7%); and *sigA* (serine protease autotransporter toxin SigA) was present in one strain from the primary inlet of the Baix Llobregat WWTP (0.7%).

The *clbA* (colibactin synthesis, linked to colorectal cancer) gene was detected in three strains (2%), one at the primary inlet of Baix Llobregat WWTP, and two at the Gavà-Viladecans WWTP, at the primary inlet and the secondary IFAS outlet, respectively.

**Fig 3:**
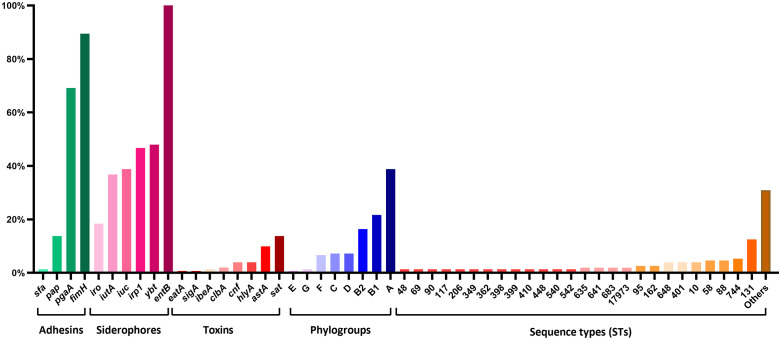
Percentage of the virulence factor genes, phylogroups and STs identified among the *E. coli* strains.

ExPEC-associated VFGs were also prevalent, particularly those linked to uropathogenic *E. coli* (UPEC). The most frequent was *sat* (secreted autotransporter toxin Sat), detected in 21 strains (13.8%). The *hlyA* (α-hemolysin) and *cnf1* (cytotoxic necrotizing factor) genes co-occurred in six strains (3.9%), including one from the advanced tertiary outlet from the Baix Llobregat WWTP. In comparison, *ibeA* (invasion-associated protein linked to newborn meningitis) gene was present in two strains (1.3%), one from the IFAS outlet from the Gavà-Viladecans WWTP, and the other from the advanced tertiary outlet from the Baix Llobregat WWTP. The e*ltA*, *aggR* and *stxA1* genes were absent in all strains (Fig 3). No toxin genes were detected at the DWTP.

All results from WWTPs and the DWTP, including all sampling points, are compiled in Table 2.

#### Integrases.

A total of 79 strains (52%) harbored integrases. *IntI1* was identified in 78 (51.3%), and *intI3* in six (3.9%). Five of six *intI*3-positive strains simultaneously carried *intI1.*

### Biofilm Formation

Strains were classified according to the criteria of Stepanović *et al*. (24), as follows: non-biofilm formers (OD ≤ 0.118), weak biofilm formers (0.118 < OD ≤ 0.237), moderate biofilm formers (0.237 < OD ≤ 0.473), and strong biofilm formers (OD > 0.473).

**Fig 4:**
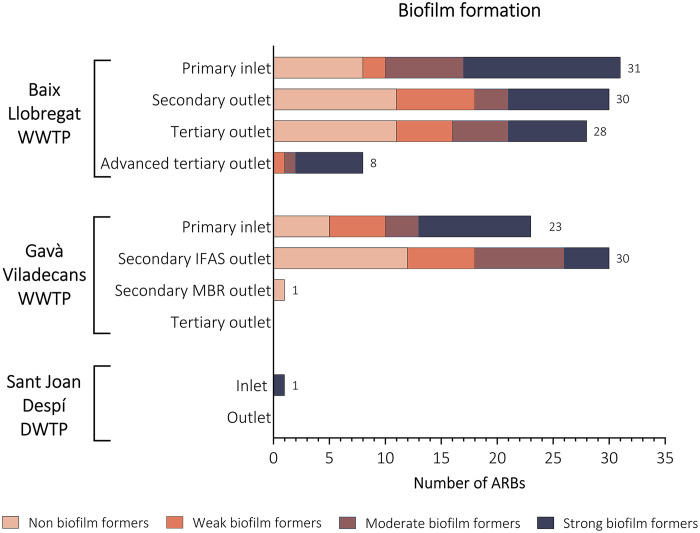
Biofilm formation classification of *E. coli* strains across treatment stages in the WWTPs and the DWTP.

Overall, 48 strains (31.6%) were non-biofilm formers, 26 (17.1%) weak, 27 (17.8%) moderate, and 51 (33.6%) strong biofilm producers (Fig 4).

At the Baix Llobregat WWTP, over 70% of strains formed biofilms, with the highest proportion of strong biofilm producers at the primary inlet. All eight strains from the final treatment stage formed biofilm, six of which were strong producers.

At the Gavà-Viladecans WWTP, nearly 67% of strains formed biofilms, with the highest proportion of strong biofilm producers predominating at the primary inlet. 60% of strains from the secondary IFAS outlet were biofilm formers, whereas the single strain from the secondary MBR outlet was non-biofilm forming. The DWTP inlet strain was classified as a strong biofilm producer.

### Statistical analyses

Statistical associations between variables were explored using logistic regression models with Firth’s penalization to reduce small-sample and sparse-data bias, and the most relevant findings are summarized below.

A strong association was observed between the presence of sulfonamide resistance genes and QACs tolerance genes (p < 0.001). The presence of integrase genes increased the likelihood of carrying sulfonamide resistance genes by approximately tenfold and QACs tolerance genes by 5.2-fold. These associations remained significant after adjustment in multivariate logistic regression models. Integrase genes also showed strong associations with other ARGs and HMTGs, including those conferring resistance to aminoglycosides, phenicols, trimethoprim, and mercury.

ESBL-producing strains harboring *bla*_CTX-M_ carried a significantly higher number of VFGs than non-ESBL strains (mean ± SD = 4.8 ± 2.3 vs. 3.9 ± 1.5; p = 0.0048). These strains were 2.97 to 20.2 times more likely to carry specific VFGs such as *hlyA*, *cnf1*, *sat*, *clbA*, *iuc/iut*, and *ybt* (yersiniabactin). Multivariate analysis confirmed the independent association with the *sat* gene. Conversely, strains carrying the type I fimbria gene were significantly less likely to harbor *bla*_CTX-M_ genes, and this inverse association persisted after multivariate adjustment.

Strains belonging to phylogroup A carried significantly fewer VFGs than those from other phylogroups (mean ± SD = 3.1 ± 1.0 vs. 4.8 ± 1.9; p < 0.0001). Statistically significant inverse associations were identified for *sat*, *iro*, *iuc/iut*, *ybt* and *pap*. In contrast, phylogroup A strains were 2.3- to 8.9-fold more likely to carry HMTGs associated with copper, silver, mercury, and tellurium.

On the other hand, phylogroup B2 strains carried significantly more VFGs than non-phylogroup B2 strains (mean ± SD = 7.0 ± 1.4 vs. 3.6 ± 1.2; p < 0.0001), and were 5.3 to 87.7 times more likely to carry specific VFGs such as *hly*, *cnf1*, *ibeA*, *clb*, *iuc/iut*, *ybt*, *pap*, *sfa* and *pgaA*, while showing a lower prevalence of HMTGs (mean ± SD = 1.2 ± 0.5 vs. 1.9 ± 1.1; p = 0.0007). Negative associations were observed with copper, silver, and mercury tolerance genes.

Similarly, high-risk clones carried a significantly higher number of VFGs than non-high-risk clones (mean ± SD = 4.8 ± 1.8 vs. 3.2 ± 1.4; p < 0.0001). These clones were 3.2 to 7.0 times more likely to harbor VFGs such as *astA*, *sat*, *iuc/iut*, *ybt*, *pap*, and *pgaA.* Multivariate analyses confirmed significant independent associations with *ybt* and *pgaA*. Conversely, high-risk clones carried fewer HMTGs than non-high-risk clones (mean ± SD = 1.6 ± 1.0 vs. 2.0 ± 1.1; p = 0.0144), with significant negative associations for copper and silver tolerance genes.

Co-occurrence analyses between biocide or HMTGs and ARGs revealed important patterns. Strains carrying mercury tolerance genes were more likely to harbor macrolide, phenicol and sulfonamide resistance genes. Copper tolerance genes were associated with phenicol and quinolone resistance genes, while silver tolerance genes showed similar associations with phenicol and quinolone resistance genes. Finally, the tolerance genes of QACs were significantly associated with sulfonamide, macrolide, phenicol, and trimethoprim resistance genes.

The detailed statistical results are available in the Supplementary Material 1 and Supplementary material 2.

## Discussion

WWTPs effectively remove suspended solids and fecal contamination, producing effluent that meets discharge or reuse standards. However, they are increasingly recognized as potential hotspots for AMR emergence, persistence, and spread. Since WWTPs are not specifically designed to eliminate ARB or ARGs, monitoring critical bacterial communities such as E. coli clones at each treatment stage is essential to understand their role in promoting AMR dissemination [26]. Pathogenic bacteria that resist antibiotics and water treatment processes represent a growing challenge for public and environmental health [27].

Our research shows that while WWTPs reduce overall bacterial loads, some MDR and high-risk *E. coli* clones persist through treatment, including in reclaimed water from Baix Llobregat WWTP. This water is commonly destined for agriculture, landscape irrigation, or environmental release [28]. This persistence is important under conditions of water scarcity, where reuse is necessary but may unintentionally promote AMR spread across human, animal, and environmental sectors.

A key observation was the superior performance of the Gavà-Viladecans WWTP, where the MBR technology substantially reduced the number of ARBs. Unlike conventional tertiary treatment at Baix Llobregat, MBR combines membrane filtration with a suspended biological reactor, creating a more reliable barrier to ARBs and ARGs [29]. In addition, the recovery of MDR strains at the advanced tertiary outlet, particularly during summer sampling periods, may suggest a seasonal effect on bacterial survival. Warmer temperatures, higher sunlight, and increased agricultural water use during summer months have been associated with higher AMR loads in effluents and downstream environments, as reported in some studies [28].

Recent policy measures, including the Spanish Royal Decree 1085/2024 on water reuse [30], mark important progress by aligning national practices with EU legislation. However, their success depends on rigorous implementation and comprehensive monitoring. This decree does not explicitly address AMR, virulence factors, or high-risk clones, all of which were detected in the reclaimed water of this study. Expanding monitoring frameworks to include these parameters and co-selective agents would substantially improve risk management and may enhance public health protection.

Similar to previous global studies [31–33], we observed the persistence of MDR *E. coli* along the water treatment plants. However, only one MDR strain was recovered at the DWTP inlet and none were detected at the drinking water outlet.

In Spain, the most prevalent ESBLs are CTX-M-15 (belonging to the CTX-M-1 group) and CTX-M-27 (belonging to the CTX-M-9 group) [34]. Both CTX-M groups are the most prevalent ESBLs found in our study. In the case of carbapenemases, our data also correlate with the most prevalent carbapenemases produced by *E. coli* reported in Spain, being OXA-48 like, VIM, NDM, and KPC [35]. Thus, these findings suggest that wastewater surveillance may be a good source for epidemiological studies, because it reflects the circulating ARGs in the area under study and, even, at country level.

The presence of ExPEC and DEC in treated wastewater [26,36,37] may suggest a potential risk associated with water discharge and reuse [38,39]. In our study, all 37 strains isolated from reclaimed water harbored at least one VFG associated with ExPEC or DEC pathotypes. In addition, consistent with our results, a study conducted in Brazil reported that ESBL-producing *E. coli* strains from commercialized chicken carcasses were 1.40 times more likely to harbor three to five ExPEC-associated VFGs compared to non-ESBL strains. Similarly, a study comparing cefotaxime-resistant *E. coli* from wastewater and clinical sources found overlapping virulence and ESBL genes, with over 30% of environmental strains carrying multiple ExPEC-associated VFGs [40]. These findings indicate that non-clinical sources can also harbor ESBL-producing E. coli carrying clinically relevant VFGs, reinforcing the idea that these environments may serve as potential pathways for the spread of pathogenic, MDR *E. coli* clones.

TET resistance was the most prevalent, accounting for the 71.1% of the strains, in comparison with other antibiotics such as CHL, with 40.1% of resistance. This difference likely reflects both historical and current antimicrobial use patterns. TET remains widely used in human and veterinary medicine worldwide, generating sustained selective pressure that favors the maintenance and dissemination of *tet* genes in environmental bacterial populations. In Spain, TET is included in the category D (“Prudent use”) of the National Plan Against Antibiotic Resistance (PRAN) classification and remains among the most frequently used antibiotics in both primary care and hospitals in Spain. In contrast, CHL use has been largely restricted in Europe due to toxicity concerns, while related phenicols such as florfenicol are primarily used in food-producing animals. Although phenicol resistance genes such as *floR* remain associated with mobile genetic elements (MGEs) and livestock production systems, the lower selective pressure exerted by these compounds may contribute to their lower prevalence compared with TET resistance.

The detection of the *mcr-9.1* gene in a carbapenemase-producing strain that remained phenotypically susceptible to colistin highlights the existence of cryptic reservoirs of last-resort antimicrobial resistance determinants. Although the gene was not phenotypically expressed under the conditions tested, its presence represents a potential clinical concern, as environmental or therapeutic selective pressures may induce its expression through regulatory mechanisms such as the QseBC two-component system. Activation of these pathways could ultimately lead to the emergence of phenotypic resistance to colistin [41–42]. The detection of *mcr* genes in environmental *E. coli* is significant, since *E. coli* may act as a reservoir and facilitate their spread. Since their first description, *mcr* genes have been identified in many human pathogens, livestock, companion animals, wildlife and environmental sources, including soil and water. Given the critical importance of colistin as a last-resort antibiotic for the treatment of infections caused by multidrug-resistant Gram-negative bacteria, ongoing surveillance of the *mcr* genes in clinical and environmental contexts remains imperative [43].

Our data suggest that co-selection mechanisms may help maintain AMR in treatment water systems. Frequent detection of integrase genes, biocide tolerance genes, and HMTGs suggests that non-antibiotic contaminants like arsenic, mercury, copper, and QACs may exert selective pressures. This supports previous evidence that metals and disinfectants in aquatic environments co-select for AMR [44–46]. The strong association between class 1 integrase genes, QACs, and sulfonamide resistance genes (p < 0.001) demonstrates the synergistic action of both antibiotic and non-antibiotic selective pressures. It reinforces the central role of MGEs in the acquisition and persistence of ARGs, biocide tolerance genes and HMTGs. The high prevalence of the clinically relevant class 1 integrase supports their use as reliable indicators of anthropogenic pollution due to their consistent link to ARGs, biocide tolerance genes and HMTGs [47]. Previous studies have shown that the *qacE*Δ*1* gene is frequently located within class 1 integrons and Tn21 transposons [48], and that treated wastewater discharges increase the abundance of *intI1* and *sul1* genes downstream, confirming their utility as key markers of human-driven AMR propagation [49].

Phylogroup A strains, although lacking key VFGs, showed MDR, biofilm formation, and HMTGs, suggesting possible adaptation to aquatic habitats and long-term survival and ARG maintenance independent of direct fecal input, traits characteristic of naturalized *E. coli* strains [27,50]. Further analyses are needed to confirm their adaptation and long-term ecological role in wastewater environments. In contrast, phylogroup B2 strains carried classical ExPEC VFGs, including *hlyA*, *cnf*, *ibeA*, *sfa*, sat, and *pap*, and were often associated with ESBL genes such as *bla*_CTX-M_ and *bla*_TEM_, confirming their status as both highly virulent and MDR pathogens.

Notably, some globally disseminated high-risk clones were also identified. ST131, the dominant ExPEC lineage worldwide, accounted for 12.5% of strains and displayed extensive resistance, including to beta-lactams, aminoglycosides, tetracyclines, fluoroquinolones, sulfonamides, and chloramphenicol. Its presence in three strains isolated from reclaimed water at the Baix Llobregat WWTP may reflect both global dissemination and local persistence, consistent with earlier reports from Barcelona and other regions worldwide [15, 51].

The detection of additional high-risk clones such as ST648 and ST744, in the primary inlet, secondary outlet and tertiary outlet from the Baix Llobregat WWTP, as well as in the primary inlet and IFAS secondary outlet from the Gavà-Viladecans WWTP, highlights the presence of clinically relevant lineages in wastewater. Importantly, no high-risk *E. coli* clones were detected in drinking water, indicating no evidence of dissemination of high-risk *E. coli* clones through the drinking water treatment process during the sampling period.

The resistance and clonal profiles observed in this study appear to be similar to those commonly reported among human clinical *E. coli* isolates in the Barcelona metropolitan area closely than those described in livestock-associated populations. The predominance of ST131, CTX-M-15-producing isolates and clinically relevant carbapenemases reflects patterns frequently observed in healthcare-associated and community-acquired infections in Spain. Although livestock production may also contribute to environmental AMR dissemination, the composition of the detected high-risk clones suggests that urban and healthcare-related sources likely play a major role in shaping the resistome of these wastewater systems.

### Notable Findings into Reclaimed Water and the DWTP Inlet

Although no high-risk *E. coli* clones were detected in reclaimed water from Gavà-Viladecans, the detection of 34 MDR or high-risk *E. coli* clones in reclaimed water from the Baix Llobregat WWTP, out of a total of 152 strains analyzed, represents one of the most notable findings of this study. Overall, 36 strains were isolated from reclaimed water from the Baix Llobregat WWTP, underscoring the persistence of ARB despite advanced wastewater treatment processes. Importantly, none of these strains harbored COL resistance genes.

At the tertiary outlet, 28 strains were recovered, including 12 high-risk clones such as ST10 and ST131. More than half of these isolates exhibited biofilm-forming capacity, with phylogroup A being the most prevalent (n = 12). These isolates frequently carried ARGs, VFGs, integrases, HMTGs, and QAC tolerance genes, highlighting the persistence of MDR *E. coli* with pathogenic potential in reclaimed water.

At the advanced tertiary outlet, the final treatment step of the Baix Llobregat WWTP, eight strains, all isolated during summer sampling campaigns, were recovered. Seven were MDR, five carried integrase genes, and all demonstrated biofilm-forming ability. All isolates harbored multiple ARGs and VFGs. Of particular interest was the detection of a high-risk ST131 strain (phylogroup B2) carrying all the siderophore genes and multiple UPEC-associated virulence markers (*pap, cnf, fimH, hlyA*), as well as *ibeA* and *pgaA*. Furthermore, two additional high-risk clones (ST10 and ST93) were identified at this stage, suggesting the persistence of clinically significant and pathogenic MDR *E. coli* strains in reclaimed water from both summer samplings.

At the Sant Joan Despí DWTP inlet, only one MDR strain (ST976, phylogroup A) was detected. Despite its low prevalence, this isolate carried multiple ARGs (*bla*_EC_, *sul1, qnrB, aac(3), aadA, tetA*), HMTGs (*ars, mer*), QACs tolerance genes, integrase genes, biofilm-forming capacity, and a wide array of VFGs, including siderophores and adhesins. Importantly, *E. coli* was absent at the DWTP outlet, suggesting effective removal during drinking water treatment under the conditions evaluated in this study.

This study has a limitation that should be acknowledged. Because genomic and phenotypic analyses were restricted to *E. coli* strains recovered on agar plates containing different antibiotics, the collection was inherently enriched for ARBs. Consequently, the prevalence of ARGs, VFGs, STs, and high-risk clones reported here reflect the resistant fraction of the *E. coli* community and should not be interpreted as representative of the overall *E. coli* population present in these aquatic environments.

## Conclusions

This study is consistent with the emerging perspective that WWTPs could function as reservoirs where MDR, virulent, and globally prevalent high-risk *E. coli* clones may persist. However, only eight of the 152 resistant *E. coli* strains were detected at the advanced tertiary outlet of the Baix Llobregat WWTP, with no evidence of such strains at the Gavà-Viladecans tertiary outlet or at the Sant Joan Despí DWTP outlet. As water reuse becomes increasingly common due to global water scarcity, the monitoring of high-risk clones such as ST131, ST648, and ST744 in reclaimed water should be considered, given their relevance to both human and environmental health.

The persistence of ESBL- and carbapenemase-producing *E. coli* at specific stages of wastewater treatment suggests the importance of continuous surveillance across both clinical and environmental settings, and for continued investment in advanced treatment technologies to mitigate AMR dissemination within wastewater systems. Importantly, no resistant *E. coli* strains were found at the outlet of the DWTP during the sampling campaigns included in this study, suggesting effective removal under the conditions evaluated. To further address these risks, wastewater management strategies should integrate advanced treatment approaches, such as MBR, alongside comprehensive AMR surveillance frameworks that include not only ARGs but also VFGs, biocide tolerance genes, and HMTGs. The incorporation of these indicators in updated regulatory frameworks could contribute to protecting public and environmental health and limiting the global spread of resistant pathogens.

## Supporting information

S1 File Statistical results. (XLSX)

S2 File Relationship between resistance phenotype and genotype. (DOCX)
