# Supplementary material for: Surveillance of Escherichia coli clones and their antimicrobial resistance profiles in wastewater and drinking water treatment plants of Barcelona, Spain
Source: PLoS One. 2026 Jul 31;21(7):e0355125. doi: 10.1371/journal.pone.0355125 (PMC13426985; doi:10.1371/journal.pone.0355125)
Supplement: S2 File — (DOCX) [file pone.0355125.s002.docx]

Aminoglycosides

| Table: Aminoglycosides | | |
| --- | --- | --- |
| Independent Association Matrices (Odds Ratio, 95% Confidence Intervals, and FDR p-values) | | |
|  | AN | GM |
| **aadA** | 1.40 [0.29-9.00] (0.821) | 1.61 [0.71-3.77] (0.380) |
| **aph(3')** | 0.29 [0.03-1.60] (0.308) | 1.46 [0.67-3.23] (0.478) |
| **aph(6)-Id** | 0.16 [0.00-1.28] (0.181) | 1.22 [0.55-2.68] (0.821) |
| **aac(3)** | 1.73 [0.26-8.64] (0.538) | 552.58 [72.33-16384.00] (<0.001)*** |
| **aac(6')-Ib** | 17.36 [2.74-108.44] (0.010)* | 7.58 [1.62-47.89] (0.024)* |
| **ant(2'')-Ia** | 4.27 [0.08-50.63] (0.380) | 4.41 [0.49-54.62] (0.229) |
| **aph(4)-Ia** | 0.00 [0.00-88.29] (1.000) | Inf [0.53-Inf] (0.170) |
| **ant(3'')-Ia** | 0.00 [0.00-613.73] (1.000) | Inf [0.07-Inf] (0.380) |
| **aac6_Ib_cr5** | 5.35 [0.77-29.24] (0.153) | 3.71 [1.08-13.06] (0.103) |
| **aac(6')-lb-cr** | 4.53 [0.66-24.23] (0.170) | 3.73 [1.17-12.18] (0.081) |
| Values represent Odds Ratios (OR) [95% Confidence Intervals] and *(FDR-adjusted p-values)*. Significance levels: (*) p-adj < 0.05, (****) p-adj < 0.01, (***) *p*-adj < 0.001. Hyphen (-) indicates untested combinations. | | |

Beta-lactams

| Table: Beta lactams | | | | | | | |
| --- | --- | --- | --- | --- | --- | --- | --- |
| Independent Association Matrices (Odds Ratio, 95% Confidence Intervals, and FDR p-values) | | | | | | | |
|  | AMP | FEP | CTX | CAZ | FOX_pheno | TZP | AMC |
| **blaEC** | - | 0.00 [0.00-Inf] (1.000) | 0.00 [0.00-Inf] (1.000) | 0.00 [0.00-Inf] (1.000) | 0.00 [0.00-Inf] (1.000) | - | - |
| **TEM** | Inf [5.82-Inf] (<0.001)*** | - | - | - | - | 0.62 [0.30-1.25] (0.412) | 0.75 [0.31-1.85] (0.851) |
| **CTX-M-1** | - | 31.84 [10.47-119.63] (<0.001)*** | Inf [38.01-Inf] (<0.001)*** | 37.06 [12.33-130.73] (<0.001)*** | - | - | - |
| **blaOXA_1** | Inf [0.68-Inf] (0.315) | - | - | - | - | 8.62 [2.41-47.17] (0.001)** | Inf [4.87-Inf] (<0.001)*** |
| **bla_KPC** | Inf [0.24-Inf] (0.932) | 26.51 [3.56-1179.81] (<0.001)*** | Inf [5.17-Inf] (<0.001)*** | 8.25 [1.85-50.96] (0.010)** | 5.33 [0.42-313.82] (0.412) | Inf [2.48-Inf] (0.006)** | Inf [1.78-Inf] (0.019)* |
| **CTX-M-9** | - | Inf [2.75-Inf] (0.006)** | Inf [2.21-Inf] (0.011)* | 1.32 [0.12-9.64] (1.000) | - | - | - |
| **bla_OXA_48** | Inf [0.09-Inf] (1.000) | Inf [2.10-Inf] (0.014)* | Inf [1.69-Inf] (0.025)* | Inf [2.56-Inf] (0.009)** | - | Inf [0.84-Inf] (0.191) | Inf [0.77-Inf] (0.152) |
| **FOX_gene** | - | Inf [0.02-Inf] (1.000) | 0.00 [0.00-Inf] (1.000) | Inf [0.13-Inf] (0.816) | 0.00 [0.00-Inf] (1.000) | - | - |
| **blaDHA-1** | - | 4.43 [0.23-266.24] (0.489) | 3.59 [0.18-215.61] (0.585) | 5.38 [0.27-323.70] (0.412) | 0.44 [0.00-39.19] (1.000) | - | - |
| **blaCMY** | - | Inf [0.91-Inf] (0.116) | Inf [0.74-Inf] (0.152) | Inf [1.11-Inf] (0.081) | Inf [0.08-Inf] (1.000) | - | - |
| **blaSHV-12** | - | 4.43 [0.23-266.24] (0.489) | Inf [0.74-Inf] (0.152) | Inf [1.11-Inf] (0.081) | - | - | - |
| **blaLAP** | - | 0.00 [0.00-11.58] (1.000) | 0.00 [0.00-9.41] (0.851) | 0.00 [0.00-14.01] (1.000) | - | - | - |
| **bla_VIM** | Inf [0.02-Inf] (1.000) | Inf [0.41-Inf] (0.266) | Inf [0.33-Inf] (0.315) | Inf [0.50-Inf] (0.219) | Inf [0.08-Inf] (1.000) | Inf [0.17-Inf] (0.826) | Inf [0.03-Inf] (0.816) |
| **bla_NDM** | Inf [0.02-Inf] (1.000) | Inf [0.41-Inf] (0.266) | Inf [0.33-Inf] (0.315) | Inf [0.50-Inf] (0.219) | Inf [0.01-Inf] (1.000) | Inf [0.17-Inf] (0.826) | Inf [0.03-Inf] (0.816) |
| **blaOXA_10** | Inf [0.00-Inf] (1.000) | - | - | - | - | Inf [0.02-Inf] (1.000) | 0.00 [0.00-Inf] (1.000) |
| **blaOXA_484** | Inf [0.00-Inf] (1.000) | Inf [0.06-Inf] (0.607) | Inf [0.05-Inf] (0.677) | Inf [0.07-Inf] (0.560) | - | Inf [0.02-Inf] (1.000) | 0.00 [0.00-Inf] (1.000) |
| **blaAER** | Inf [0.00-Inf] (1.000) | - | - | - | - | Inf [0.02-Inf] (1.000) | 0.00 [0.00-Inf] (1.000) |
| Values represent Odds Ratios (OR) [95% Confidence Intervals] and *(FDR-adjusted p-values)*. Significance levels: (*) p-adj < 0.05, (****) p-adj < 0.01, (***) *p*-adj < 0.001. Hyphen (-) indicates untested combinations. | | | | | | | |

Phenicols

| Table: Phenicols | |
| --- | --- |
| Independent Association Matrices (Odds Ratio, 95% Confidence Intervals, and FDR p-values) | |
|  | CHL |
| **cat** | 7.14 [2.74-21.16] (<0.001)*** |
| **floR** | 90.75 [14.08-3747.90] (<0.001)*** |
| **cmIA** | 23.11 [3.37-995.54] (<0.001)*** |
| Values represent Odds Ratios (OR) [95% Confidence Intervals] and *(FDR-adjusted p-values)*. Significance levels: (*) p-adj < 0.05, (****) p-adj < 0.01, (***) *p*-adj < 0.001. Hyphen (-) indicates untested combinations. | |

Quinolones

| Table: Quinolones | |
| --- | --- |
| Independent Association Matrices (Odds Ratio, 95% Confidence Intervals, and FDR p-values) | |
|  | CIP |
| **qnrS** | 8.55 [2.01-77.11] (0.002)** |
| **qnrB** | 4.22 [0.58-187.30] (0.370) |
| **qnrA1** | Inf [0.07-Inf] (1.000) |
| **qepA** | Inf [0.01-Inf] (1.000) |
| Values represent Odds Ratios (OR) [95% Confidence Intervals] and *(FDR-adjusted p-values)*. Significance levels: (*) p-adj < 0.05, (****) p-adj < 0.01, (***) *p*-adj < 0.001. Hyphen (-) indicates untested combinations. | |

Sulfonamides

| Table: Sulfonamides | |
| --- | --- |
| Independent Association Matrices (Odds Ratio, 95% Confidence Intervals, and FDR p-values) | |
|  | SXT |
| **sul1** | 5.43 [2.35-13.57] (<0.001)*** |
| **sul2** | 6.17 [2.61-16.03] (<0.001)*** |
| **sul3** | 11.11 [1.66-474.40] (0.004)** |
| Values represent Odds Ratios (OR) [95% Confidence Intervals] and *(FDR-adjusted p-values)*. Significance levels: (*) p-adj < 0.05, (****) p-adj < 0.01, (***) *p*-adj < 0.001. Hyphen (-) indicates untested combinations. | |

Tetracyclines

| Table: Tetracyclines | |
| --- | --- |
| Independent Association Matrices (Odds Ratio, 95% Confidence Intervals, and FDR p-values) | |
|  | TET |
| **tetA** | 101.45 [15.81-4169.62] (<0.001)*** |
| **tetB** | Inf [4.28-Inf] (<0.001)*** |
| **tetM** | Inf [0.78-Inf] (0.063) |
| Values represent Odds Ratios (OR) [95% Confidence Intervals] and *(FDR-adjusted p-values)*. Significance levels: (*) p-adj < 0.05, (****) p-adj < 0.01, (***) *p*-adj < 0.001. Hyphen (-) indicates untested combinations. | |

Trimethoprim

| Table: Trimethoprim | |
| --- | --- |
| Independent Association Matrices (Odds Ratio, 95% Confidence Intervals, and FDR p-values) | |
|  | SXT |
| **dfr** | 341.78 [67.21-3788.02] (<0.001)*** |
| Values represent Odds Ratios (OR) [95% Confidence Intervals] and *(FDR-adjusted p-values)*. Significance levels: (*) p-adj < 0.05, (****) p-adj < 0.01, (***) *p*-adj < 0.001. Hyphen (-) indicates untested combinations. | |
